# Red Book: 2006 Report of the Committee on Infectious Diseases, 27th Edition

**DOI:** 10.3201/eid1212.061045

**Published:** 2006-12

**Authors:** Andi L. Shane

**Affiliations:** * Emory University School of Medicine, Atlanta, Georgia, USA

**Keywords:** Red Book, L.K. Pickering, C.J. Baker, S.S. Long, J.A. McMillan, Committee on Infectious Diseases

The 27th edition of the 2006 Report of the American Academy of Pediatrics (AAP) Committee on Infectious Diseases, known to most clinicians as "The Red Book," is considered the "Bible" of pediatric infectious diseases. In addition to providing an updated and exhaustive summary of the clinical manifestations, etiology, epidemiology, diagnostic tests, treatment, isolation, and control measures for >200 pediatric infectious diseases, this reference discusses a number of related topics, including management. With >350 liaisons and collaborators from the Centers for Disease Control and Prevention, the Food and Drug Administration, the National Institutes of Health, the Canadian Paediatric Society, the World Health Organization, and others, the 12-member 2004–2006 Committee on Infectious Diseases issued the current edition, which reflects the state of the art at the time of publication and is updated every 3 years.

The Red Book began as an 8-page mimeographed report assembled in 1937 by the Committee on Immunization and Therapeutic Procedures for Acute Infectious Diseases (currently the Committee on Infectious Diseases) and titled "Immunization Procedures." A revision issued in 1938 in pamphlet form was actively sought, and annual issues became more comprehensive, with the addition of 76 pages reflecting the increase in therapeutic antimicrobial drug options in the ensuing 10 years.

The current 992-page 27th edition has newly added sections, including key developments in combination vaccines, the 2006 AAP standards for child and adolescent immunization practices, parental refusal to vaccinate, updates on prevention of mosquitoborne infections, and updated information on emerging infectious diseases and pathogens, including Baylisascaris, metapneumovirus, West Nile virus, coronaviruses, pertussis, tuberculosis, and pneumococcal, meningococcal, and varicella infections. Expanded discussions of drug interactions as well as the revised American Heart Association recommendations for the prevention of bacterial endocarditis are presented. Many website resources have been added throughout the text to provide the reader with links to expanded information about the topic.

The text is organized into 5 sections with a comprehensive list of appendixes. Section 1 reviews active and passive immunization and provides a practical discussion of numerous aspects of vaccine administration, including vaccine shortages, reporting of vaccine-preventable diseases, and parental misconceptions about vaccinations. Section 2 provides recommendations for care of children in special circumstances, including the topic areas of biological terrorism, children in out-of-home child care, infection control, and medical evaluation of internationally adopted children. This section serves as a comprehensive resource for both general and infectious diseases pediatricians. The 3rd section, an alphabetical summary of infectious diseases, comprises the bulk of the text. Sections 4 and 5 address the expanding category of antimicrobial agents, therapy, and prophylaxis, including guidelines and indications for their appropriate use.

The area of infectious diseases is rapidly emerging and changing, so the guidelines presented in the current edition may have undergone updating and revision following publication. Therefore, readers are urged to monitor updated recommendations issued by the Committee on Infectious Diseases on the Red Book Online website (http://www.aapredbook.org). This site also lists errata from the current edition and allows readers to register to be notified when new errata are posted, when new policy statements are issued, and when site updates and new features are added. Readers may also register for a customized citation/keyword alert.

The 27th edition of the Red Book is a vital resource for adult and pediatric infectious disease practitioners as well as general practitioners and is considered by many the quintessential resource and reference for clinical practice.

